# Case Report: Mucinous Adenocarcinoma Arising From Congenital Ejaculatory Duct Cyst

**DOI:** 10.3389/pore.2021.528050

**Published:** 2021-03-26

**Authors:** Hua Shen, Kai Liao, Weili Wu, Gongyu Li, Shijin Chen, Nan Nan, Hongbo Yu, Hongfei Wu

**Affiliations:** ^1^ Department of Urology, BenQ Medical Center, The Affiliated BenQ Hospital of Nanjing Medical University, Nanjing, China; ^2^ Department of Pathology, BenQ Medical Center, The Affiliated BenQ Hospital of Nanjing Medical University, Nanjing, China; ^3^ Department of Radiology, BenQ Medical Center, The Affiliated BenQ Hospital of Nanjing Medical University, Nanjing, China

**Keywords:** mucinous adenocarcinoma, ejaculatory duct, ejaculatory duct cyst, ejaculatory duct tumor, ejaculatory duct adenocarcinoma

## Abstract

Herein we present a previously unreported rare case of mucinous adenocarcinoma arising from a congenital ejaculatory duct cyst. Radiographic and endoscopic examinations revealed the tumor occurred in a cyst running through the prostate. Initially, the immunohistochemical pathology results showed that it was a metastatic mucinous adenocarcinoma, but no other primary lesions were clinically evidenced. Based on the embryonic development process of the male urogenital tract, the malformation of the patient's ejaculatory duct, and the pathological examination of the resected specimen, we considered the tumor to be a primary mucinous adenocarcinoma which originating from the hypoplastic ejaculatory duct. The tumor may have developed from the foci of intestinal metaplasia from cloacal remnants during embryonic development.

## Introduction

The ejaculatory tube is composed of the excretory tube of the seminal vesicle and the vas deferens. Bilateral tubes enter into the base of the prostate and opening on both sides of the membrane of verumontanum. Ejaculatory duct cyst is rare congenital or acquired lesion, with fewer than 30 previous reports in the literature [12]. Only two cases of ejaculatory duct tumors have been reported, including one case of adenomatoid tumor of the ejaculatory duct [2] and another case of intraductal carcinoma of the prostate in the ejaculatory duct [9].

Mucinous adenocarcinomas are malignant tumor which usually occur in the gastrointestinal tract and are rare in the genitourinary tract. We report a case of primary mucinous adenocarcinoma arising from a congenital ejaculatory duct cyst, which may have been related to the cloacal remnants during embryonic development.

## Case Report

**FIGURE 1:**
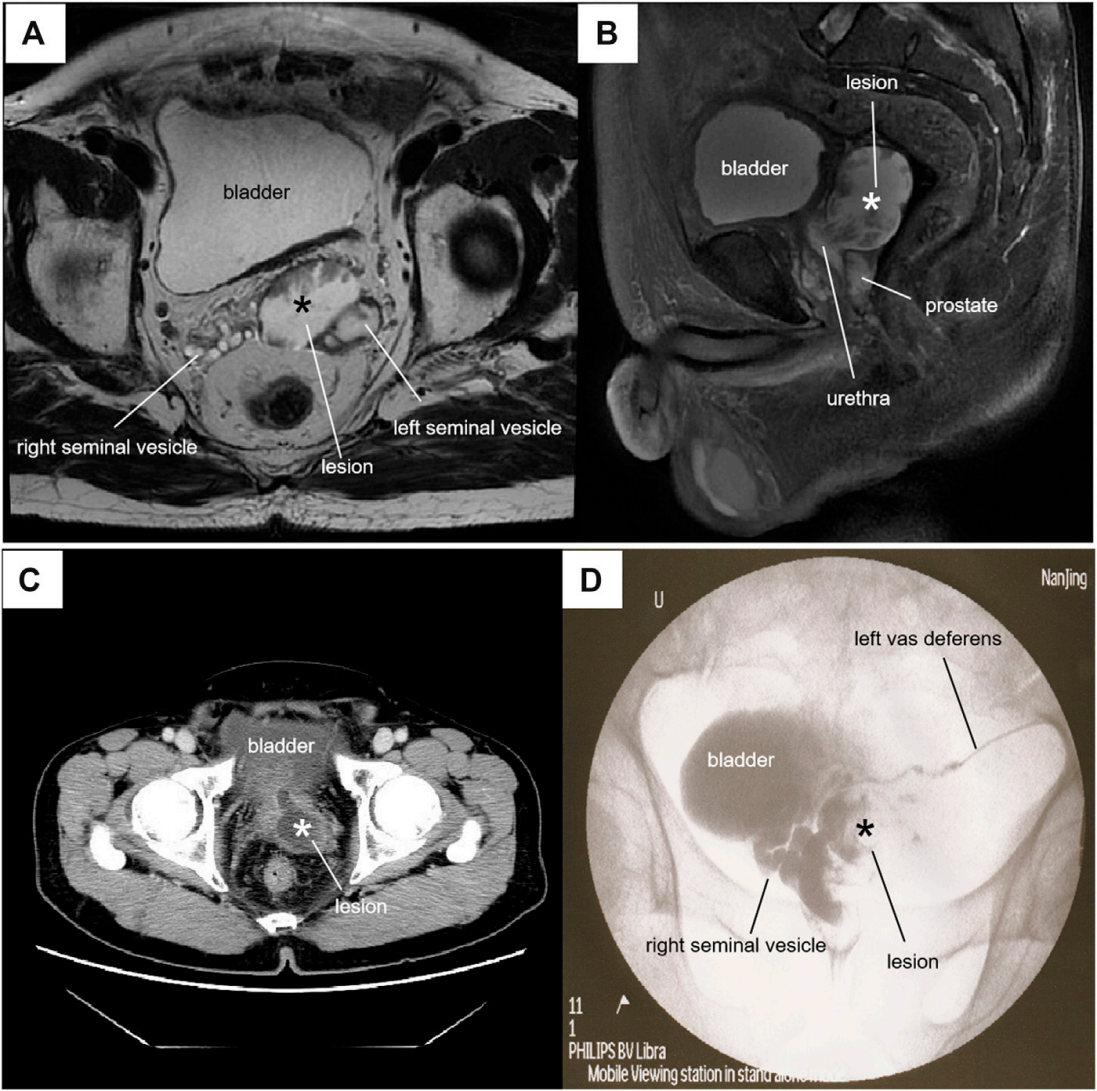
Radiographic features of the lesion*. **(A)** Axial T2-weighted MRI showed a 45 × 36×44-mm lesion with mixed signals at the left lower posterior of the bladder. **(B)** Sagittal fat-suppressed T2-weighted MRI showed that the cystic-solid lesion passed through the prostate into the posterior urethra. **(C)** Contrast-CT showed a cystic-solid mass with obvious enhancement of the cyst wall communicating with the urethra. **(D)** Vasography showed a filling defectan in the cystic structure.

**FIGURE 2:**
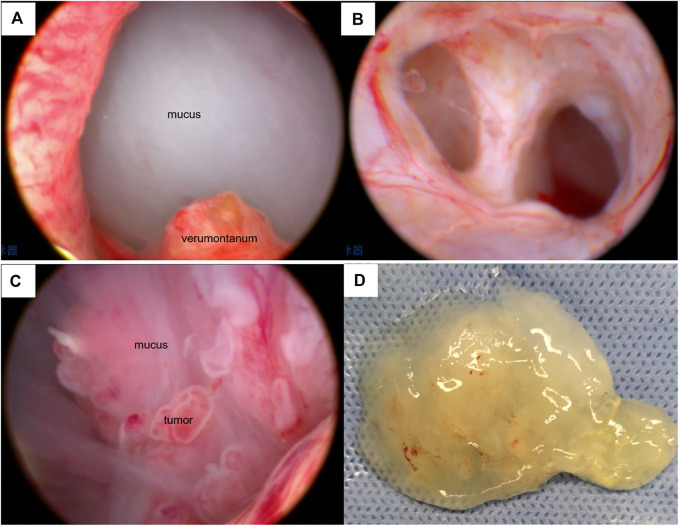
Cystoscopic features of the lesion. **(A)** Spherical mucus-like barrier in the prostatic urethra under cystoscopy. **(B)** The cystoscope entered the right seminal vesicle. **(C)** Cystoscopy showed a mucoid substance and multiple papillary neoplasms in the cystic cavity. **(D)** Jelly-like substance sucked out from the cystic cavity.

**FIGURE 3:**
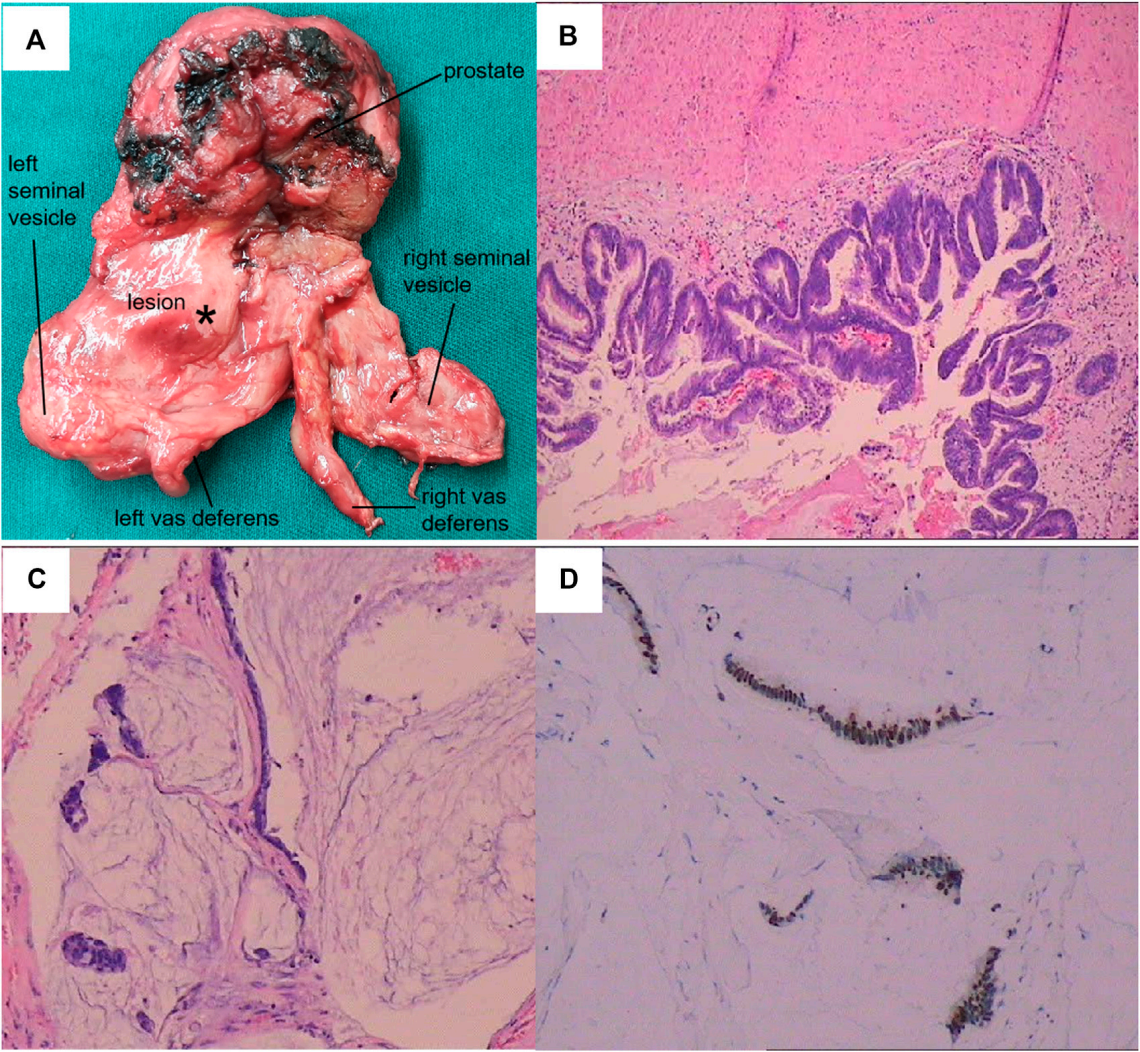
Pathological features of the lesion. **(A)** General view of the resected specimen. **(B)** Hematoxylin and eosin (HE) staining of the neoplasm (100×). **(C)** HE staining of the mucinous area (100×). **(D)** Immunohistochemistry showed strong CDX-2^+^ expression.

A 74-year-old man who was married with a son and daughter was admitted because of intermittent mucoid discharge from his urethra and difficulty voiding for 3 years. Indwelling catheterization was performed because of urinary retention. Digital rectal exanmination revealed a suspicious soft cystic mass with an unclear boundary in the upper left area of the moderately enlarged prostate. Serum prostate-specific antigen (PSA) was 4.12 ng/ml. Imaging studies (Figure 1) and an endoscopic examination (Figure 2) were subsequently performed. Magnetic resonance imaging (MRI) detected an irregular cystic-solid mass with villiform long T1 and T2 signals in the left ejaculatory duct area. Vasography showed an incomplete cyst-like structure with a filling defect under the bladder. Cystoscopy disclosed a cystic cavity filled with mucoid substance and multiple papillary neoplasms, which opened into the prostatic urethra near the top of the verumontanum. The cystoscope could be inserted into the right seminal vesicle through the urethral opening. Biopsies showed that the neoplasm was a metastatic mucinous adenocarcinoma or tubulo-villous adenoma. Immunohistochemistry revealed it to be a mucinous adenocarcinoma. All gastrointestinal tract tumor markers were normal. Computed tomography (CT) scanning showed a cystic-solid mass occupying the left ejaculatory duct area without renal agenesis. Contrast agent revealed an obviously enhanced cyst wall and a papillary lesion at the junction of the sigmoid colon and rectum. Colonoscopy revealed a 2.0-cm polypoid mass in the proximal rectum. An endoscopic polypectomy was performed and the pathological result was a tubulo-villous adenoma. A radical prostatectomy and seminal vesiculectomy were then performed. The pathological diagnosis was adenocarcinoma with 55% mucinous adenocarcinoma (enteric-type). No lipochrome pigment granules were oberved in the tumor. Immunohistochemistry showed PSA (−), P504S (+), 34βE12 (+/−), P63 (−), ERG (−), CK7 (+), CK20 (+), CDX-2 (3+), SATB-2 (−), CEA (−), AR (−), Ki-67 (40%+), PAX2 (−), PAX8 (−) and MUC6 (2%+) (Figure 3).

## Discussion

Mucinous adenocarcinomas are a special pathological type of adenocarcinoma, in which >50% of the lesion is composed of pools of extracellular mucin [3]. Of all colorectal cancers, 5–15% meet the criteria for mucinous adenocarcinoma [4]. Mucinous adenocarcinomas of the prostate, in which >25% of the lesion is extravasated mucin, are one of the rarest morphological variants of all prostatic carcinomas, with an incidence of 0.21–0.43% [1]. The more common metastatic mucinous adenocarcinoma of the prostate is often secondary to colorectal, bladder or urethral tumors [8]. Mucinous adenocarcinomas of the ejaculatory duct have not previously been reported.

This case was pathologically confirmed as a mucinous adenocarcinoma, occurring in a cystic structure with invasion of the left seminal vesicle and vas deferens. Three-dimensional radiological reconstruction and histological examination revealed no normal ejaculatory duct was found in the left prostate lobe. The cyst, which was located at the left lower posterior of the bladder, passed through the prostate and connected to the right seminal vesicle through the prostatic urethra (Figure 1). We therefore considered this cystic structure to be a dysplastic left ejaculatory tube. Negative PSA, CEA and AR expressions excluded primary prostate or seminal vesicle adenocarcinoma. Although the tumor could be detected in the left seminal vesicle, and P504S was positively expressed in the seminal vesicles and vas deferens, the primary seminal vesicle carcinoma showed mostly CK7+ and CK20− immunostaining [7]. In this case, the tumor was CK20^+^, PAX2^−^ and PAX8^−^, indicating that it was not derived from the mesonephric or paramesonephric duct. Thus, the tumor likely did not arise from the seminal vesicle or vas deferens. Strong CDX-2^+^ expression indicated that it was either metastatic colorectal mucinous adenocarcinoma or intestinal metaplasia, but no clinical evidence was found to support colorectal tumor metastasis, other than a rectal tubulo-villous adenoma.

**FIGURE 4:**
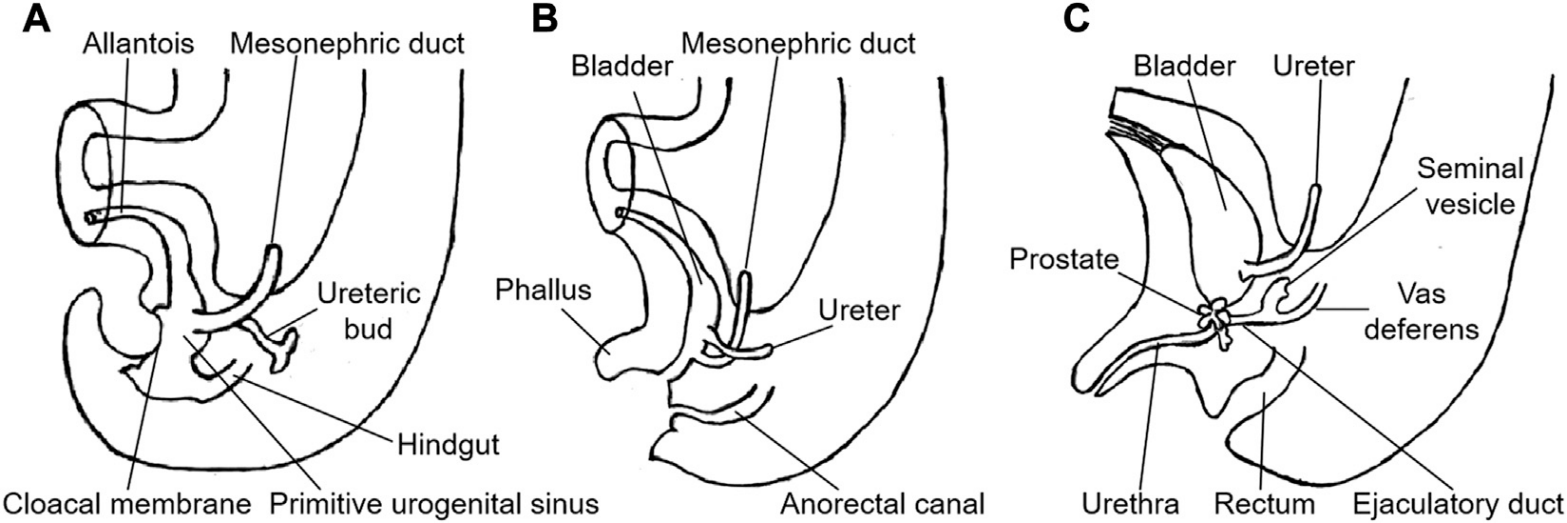
Embryonic development of the male urogenital tract. **(A)** fifth week. **(B)** eighth week. **(C)** 13th week. (Redrawn from Langman's Medical Embryology. 13th Ed. TW Sadler. 2015: 259).

The cloaca is the common compartment in the urogenital and anorectal channels in the 5-week-old human embryos. During weeks six to seven of embryonic development, the urorectal septum subdivides the cloaca into two separate parts [5]. The allantoic canal in the upper part of the primitive urogenital sinus forms the bladder and urethra, while the prostate develops from the lower urogenital sinus. The mesonephric duct is included in the vesicourethral canal within the urogenital sinus. Between weeks 12 and 13 of fetal development, the mesonephric duct develops and forms the epididymal duct, vas deferens and seminal vesicle under the action of testosterone. The ampulla of the vas deferens and seminal vesicle join posteriorly and superiorly to the prostate to form the ejaculatory duct, which opens into the prostatic urethra (Figure 4) [13]. In this case, the cystic ejaculatory duct developed a mucinous adenocarcinoma, which should have occurred in the colorectum. Although the tumor's precise pathogenesis was unknown, it may have developed from the foci of intestinal metaplasia from cloacal remnants, which is similar to the genesis of primary vaginal adenocarcinoma with mucinous-enteric differentiation [14].

Interestingly, the initial pathological diagnosis suggested that the neoplasm in the cyst was a tubulo-villous adenoma, which also occurred in the patient's rectum. Of colorectal adenocarcinomas, 95% develop from adenomas, which are classified as villous, tubular or tubulo-villous. The latter two are highly correlated with malignancy (including mucinous adenocarcinoma) because they contain villous tissue [6]. Several reports have also described villous adenomas of the urinary tract in which glandular epithelial lesions arose in the urothelial-lined urinary tract [10, 11]. Cloacal tissue may remain in the urinary tract, with the potential to give rise to glandular epithelial neoplasms. In this case, the tubulo-villous adenoma may have occurred in the ejaculatory duct cyst with cloacal remnants and developed into mucinous adenocarcinoma after stimulation by harmful substances in the urine retained in the cyst.

The patient underwent radical prostatectomy and seminal vesiculectomy. MRI performed 6 months postoperation showed normal signaling of the vesicourethral anastomosis area and no enlarged lymph node was detected in the pelvis. However, 15 months postoperation, the patient experienced difficulty urinating. Cystoscopic examination revealed neoplasms at the vesicourethral anastomosis. The sample obtained via transurethral resection was pathologically confirmed to be mucinous adenocarcinoma. Tumor-cell seeding through the mucus secreted by the tumor might have been the main cause of recurrence. Chemotherapy was recommended.

## References

[1] Bohman K. D. Osunkoya A. O. (2012). Mucin-producing tumors and tumor-like lesions involving the prostate: a comprehensive review. Adv. Anat. Pathol. 19 (6), 374–387. 10.1097/PAP.0b013e318271a361 23060063

[2] Fan K. Johnson D. F. (1985). Adenomatoid tumor of ejaculatory duct. Urology 25, 653–654. 10.1016/0090-4295(85)90307-3 4012964

[3] Hamilton S. R. Aaltonen L. A. (2000). World health organization classification of tumours (Lyon, France: IARC Press).

[4] Kang H. O'Connell J. B. Maggard M. A. Sack J. Ko C. Y. (2005). A 10-year outcomes evaluation of mucinous and signet-ring cell carcinoma of the colon and rectum. Dis. Colon Rectum 48, 1161. 10.1007/s10350-004-0932-1 15868237

[5] Kruepunga N. Hikspoors J. P. J. M. Mekonen H. K. Mommen G. M. C. Meemon K. Weerachatyanukul W. (2018). The development of the cloaca in the human embryo. J. Anat. 233 (6), 724–739. 10.1111/joa.12882 30294789 PMC6231168

[6] Morson B. C. Sobin L. H. (1976). “Histological typing of intestinal tumours,” in International histological classification of tumours. No. 15. Geneva: World Health Organization, 69.

[7] Ormsby A. H. Haskell R. Jones D. Goldblum J. R. (2000). Primary seminal vesicle carcinoma: an immunohistochemical analysis of four cases. Mod. Pathol. 13 (1), 46–51. 10.1038/modpathol.3880008 10658909

[8] Osunkoya A. O. Nielsen M. E. Epstein J. I. (2008). Prognosis of mucinous adenocarcinoma of the prostate treated by radical prostatectomy: a study of 47 cases. Am. J. Surg. Pathol. 32 (3), 468–472. 10.1097/PAS.0b013e3181589f72 18300802

[9] Sanchez-Salazar A. J. Basler J. W. Nicolas M. M. (2010). Intraductal carcinoma of the prostate in the ejaculatory duct. Int. J. Surg. Pathol. 18, 298–299. 10.1177/1066896910364534 20444733

[10] Seibel J. L. Prasad S. Weiss R. E. Bancila E. Epstein J. I. (2002). Villous adenoma of the urinary tract: a lesion frequently associated with malignancy. Hum. Pathol. 33 (2), 236–241. 10.1053/hupa.2002.31293 11957151

[11] Wang J. Manucha V. (2016). Villous adenoma of the urinary bladder: a brief review of the literature. Arch. Pathol. Lab Med. 140 (1), 91–93. 10.5858/arpa.2014-0198-RS 26717061

[12] Wang S. Chen S. W. Liu W. Zhu H. J. Jiang H. Wang Y. B. (2007). Laparoscopic excision of extraprostatic ejaculatory duct cyst. Andrologia 39, 81–86. 10.1111/j.1439-0272.2007.00768.x 17683467

[13] Wein A. J. (2012). Campbell walsh-urology. 10th Edn. Philadelphia, PA: Saunders Elsevier, 1012–1013.

[14] Werner D. Wilkinson E. J. Ripley, D. Yachnis A. (2004). Primary adenocarcinoma of the vagina withmucinous-entericdifferentiation: a report of two cases with associated vaginal adenosis without history of diethylstilbestrol exposure. J. Low. Genit. Tract Dis. 8 (1), 38–42. 10.1097/00128360-200401000-00009 15874835

